# Determination of Lactoferrin Using High-Frequency Piezoelectric Quartz Aptamer Biosensor Based on Molecular Bond Rupture

**DOI:** 10.3390/molecules29235699

**Published:** 2024-12-02

**Authors:** Haizhi Wu, Shihui Si, Zheng Li, Jiayou Su, Shangguan Jia, Hao He, Chengcheng Peng, Tongqiang Cheng, Qian Wu

**Affiliations:** 1 Hunan Provincial Institute of Product and Goods Quality Inspection, Changsha 410007, China; whzh861028@126.com (H.W.); lizheng8882024@126.com (Z.L.); shangguanjia87@163.com (S.J.); 18673272562@163.com (H.H.); pcc1019@126.com (C.P.); hhctq@126.com (T.C.); 13278867896@163.com (Q.W.); 2 College of Chemistry and Chemical Engineering, Central South University, Changsha 410083, China

**Keywords:** quartz crystal microbalance, molecular bond crack, lactoferrin, aptamer

## Abstract

In this study, an aptamer biosensor for detecting lactoferrin (LF) was developed using piezoelectric quartz-induced bond rupture sensing technology. The thiol-modified aptamer I was immobilized on the gold electrode surface of the quartz crystal microbalance (QCM) through an Au-S bond to specifically bind LF. It was then combined with aptamer–magnetic beads to amplify the mass signal. The peak excitation voltage was 8 V at the resonance frequency for the 60 MHz gold-plated quartz crystal. When the molecular bond cracking process occurred, the aptamer–magnetic beads combined on the surface of the piezoelectric quartz were removed, which resulted in an increase in quartz crystal resonance frequency. Therefore, the specific detection of LF can be realized. Under optimized experimental conditions, the linear range for LF was 10–500 ng/mL, the detection limit (3σ) was 8.2 ng/mL, and the sample recoveries for actual milk powder samples ranged from 97.2% to 106.0%. Compared with conventional QCM sensing technology, the signal acquisition process of this sensing method is simple, fast, and easy to operate.

## 1. Introduction

LF is an 80 kDa protein in biological fluids, consisting of 689 amino acids and containing 17 intramolecular disulfide bonds (S-S). Its three-dimensional structure contains two similar lobes with certain biological functions, including broad-spectrum antifungal, antibacterial, and anti-inflammatory activity [1,2]. Due to its importance in promoting the immune system in infants and its easy absorption [3], LF has been widely used in infant formula milk powder as a food additive. It can improve the intestinal flora of infants, promote iron absorption, enhance immune capacity, and reduce the lipid oxidation reaction of milk powder [4]. In 2000, LF was designated by the U.S. Food and Drug Administration (FDA) as a recognized safe food additive that can be used in different categories of foods. In 2012, the European Commission approved the addition of LF to certain foods to enhance health functions. The US FDA allows for a maximum amount of LF to be used of between 100 and 400 mg/100 g. The maximum allowable use in the EU is between 80 and 3000 mg/100 g. The current trend in infant formula manufacturing is to provide functional effects similar to breast milk, and LF is an important component of breast milk composition. In order to better study LF and its related products, it is imperative to establish an effective detection method [5]. Therefore, it is important to develop LF detection methods with high sensitivity and high specificity.

The detection of LF has been made possible by a variety of techniques over the years, including high-performance liquid chromatography (HPLC) [6], high-performance liquid chromatography via tandem mass spectrometry (HPLC-MS/MS) [7], polyacrylamide gel electrophoresis (PAGE) [8], spectrophotography (SPF) [9], enzyme-linked immunosorbent assay (ELISA) [10,11], and the electrochemical biosensing method (ERM) [12,13,14]. However, due to the intricate composition of the dairy matrix, the sample pretreatment using HPLC and HPLC-MS/MS incurs substantial costs. When PAGE was used, the acrylamide monomer used was highly toxic to the skin and nervous system. At the same time, the reproducibility of gel polymerization is poor due to the influence of polymer concentration, temperature, pH value, light, and other factors. SPF is fast and simple, but the detection accuracy cannot meet the quantitative needs of LF in dairy products [15]. The ELISA study includes a double-antibody sandwich and competitive inhibition, with corresponding enzyme-linked immunodetection kits for the LF of several common matrix sources [16,17]. However, this method requires multiple dilutions of the sample before sample measurement. The process requires washing and incubation steps, so it takes a long time and has poor repeatability. The ERM have the advantages of high sensitivity, easy operation, and fast response time [18].

QCM has the advantages of simple structure, low cost, and no labeling for sensor detection. It is widely used as a high-precision quality sensor for the detection of protein molecules [19]. However, conventional QCM sensing techniques are usually detected in a way that increases the crystal surface mass, resulting in a frequency decrease. Therefore, the flow injection method is generally used [20]. The detection process usually exists in some interference factors, such as the production of bubbles in the circulation pool and non-specific binding.

Considering the advantages of sensor-based methods and the disadvantages of conventional QCM sensing techniques, this study developed a high-frequency piezoelectric quartz aptamer biosensor to detect LF. The method is based on the amplitude shear oscillation of piezoelectric quartz crystal, which provides a novel approach for mass biosensing technology. Its principle lies in increasing the excitation voltage of the quartz crystal to enhance shear motion force (momentum). Once a certain threshold is reached, repeated bond cracking and rejection occur on the surface of macromolecules. The sensing signal can be obtained through frequency appreciation or electric noise generated by the piezoelectric effect of the quartz crystal [21]. This method not only overcomes limitations associated with conventional QCM methods but also proves more suitable for large-volume sample testing. At the same time, aptamer APT (aptamer) is selected. Compared with ordinary antibodies, aptamer has the advantages of easy synthesis, purified modification, stable properties, and simple fixation steps [21].

**Figure 1:**
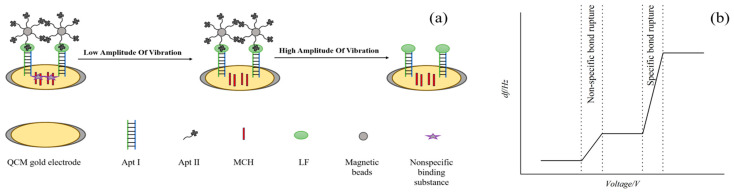
Schematic illustration of high fundamental piezoelectric quartz aptamer biosensor for the detection of LF based on molecular bond rupture technique (**a**) Δf of QCM under low/high voltage (**b**).

In this study, a 60 MHz gold-plated piezoelectric crystal served as the mass-sensing element. Employing the double-aptamer sandwich approach, aptamer I was anchored to the QCM electrode via the Au-S bonds to capture LF and was paired with the magnetic bead-bound aptamer II to enhance mass detection. The biosensor developed in our laboratory for molecular bond cracking was utilized to adjust the excitation voltage potential applied on quartz. When a low amplitude voltage is applied, it causes the weak bond between the non-specific binding substance and the gold electrode to fracture. Conversely, when a high amplitude voltage is applied, it breaks the strong binding bond between the aptamer II–magnetic bead complex and LF, enabling specific detection of LF. During the bonding process, we obtained the response signal (Δ*f*) related to the surface mass change by measuring the difference frequency signal (*df*) of QCM. The magnitude of Δ*f* corresponds directly to the mass of detached aptamer II–magnetic bead complex, which is proportional to LF concentration. And the sensor’s detection mechanism is depicted in Figure 1.

## 2. Results and Discussion

### 2.1. Signal Stability of QCM Molecular Bond Rupture Sensing System

**Figure 2:**
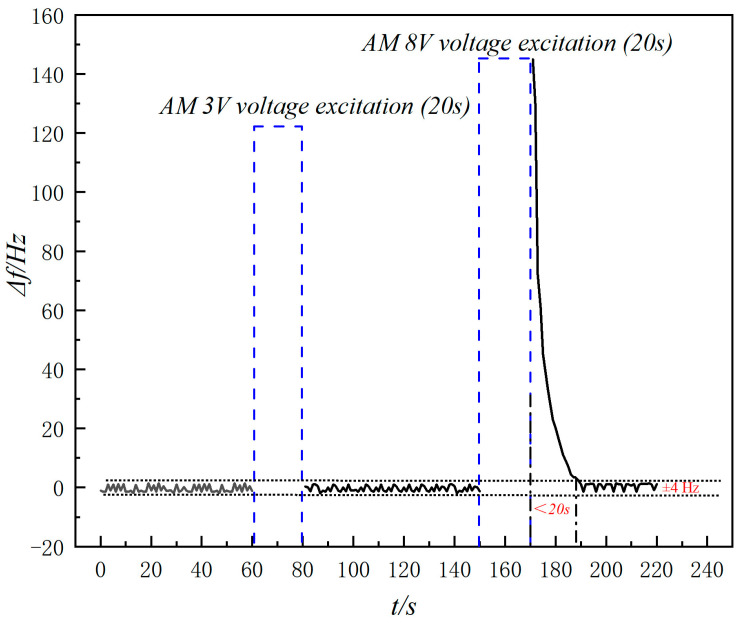
Instrument stability under gas phase.

The stability of the differential frequency circuit signal for the aptamer I-modified QCM in air is illustrated in Figure 2. After a 20 s excitation at a voltage amplitude of 3 V, the system regained stability within 20 s. Similarly, after 20 s of excitation with a voltage amplitude of 8 V, stability was regained within 20 s. Throughout the bond rupture process, the differential frequency signal variation was below ±4 Hz. In further study, the amplitude control voltage was maintained at 8 V (peak) for 20 s.

### 2.2. Characterization of Magnetic Beads Aptamers

The Zeta potential of bare magnetic beads is −65.3 mV, while the Zeta potential of magnetic beads coupled with aptamer II drops to −53.9 mV. This result can be attributed to the negatively charged adapter occupying the carboxyl site on the surface of the magnetic beads [22], indicating the successful synthesis of the magnetic bead–aptamer.

### 2.3. Feasibility of Sensor Detection of LF

**Figure 3:**
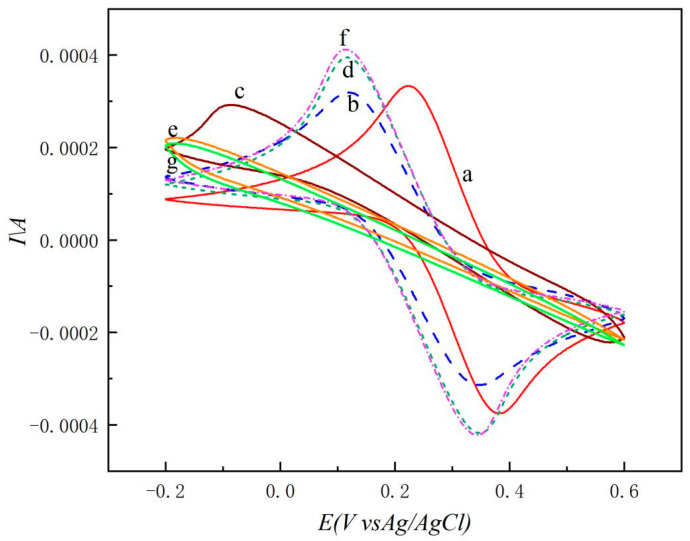
Feasibility test of sensor. Cyclic voltammetry (CV) curves of feasibility test of sensor: (a) QCM Au chip; (b) 3 μmol/L aptamer I; (c) 1 mmol/L MCH; (d) 100 ng/mL LF (30 °C); (e) 1 μmol/L aptamer II-magnetic beads (30 °C); (f) after experiment of bond rupture; (g) 1 μmol/L aptamer II-magnetic beads (after experiment of bond rupture).

The potential of amplitude-modulated QCM for facilitating molecular bond cleavage between LF and the aptamer was assessed using electrochemical detection signals. As shown in Figure 3, symmetrical redox peaks of [Fe(CN)_6_]^3−^/^4−^ are observed on the bare quartz gold electrode (curve a). After modifying with aptamer I, the peak current response decreases and the peak position shifts. This is due to the negative charge of the nucleic acid backbone, which hinders the electron transfer process of [Fe(CN)_6_]^3−^/^4−^ (curve b). Blocking the active site with MCH further reduces the peak current (curve c). When LF specifically binds to the aptamer and is fixed on the electrode, the peak current signal significantly increases due to the promotion of electron transfer on the electrode surface by Fe^2+^ in LF (curve d) [23]. Binding LF to the magnetic bead–aptamer II significantly hinders electron transfer on the electrode surface, resulting in a decreased peak current signal (curve e). After the bond rupture experiment, the peak current increases, equivalent to the current value before adding the magnetic bead–aptamer (curve f). By comparing curve f (after the detachment of magnetic bead–aptamer II) with curve d (before the addition of magnetic bead–aptamer II), we can find that curve f is very close to curve d, which indicates that the bond rupture only affected the binding of the LF–aptamer II–magnetic bead complex. This indicates that the detached material after bond cleavage is the magnetic bead–aptamer II, which is attached to the outer layer compared to the LF–magnetic bead–aptamer II. The magnetic bead, having a larger mass, detaches more easily from the crystal surface during the bonding process. After bond cleavage, the magnetic bead–aptamer II solution was applied to the crystal and subjected to electrochemical detection, showing a decrease in peak current (curve g). By comparing curve g with curve e (after the addition of magnetic bead–aptamer II), it can be observed that curve g is very close to curve e, which indicates that the binding of other bonds on the sensor was not destroyed after the bond rupture. This indicates that the aptamer magnetic bead remained on the crystal surface due to binding with LF, confirming that the detached part was solely the magnetic bead–aptamer complex. Amplitude modulation bond rupture experiments were performed using a QCM sensor. After the first bond rupture experiment, the frequency change value significantly increased, and the aptamer II magnetic beads on the surface QCM crystal were successfully detached. A second bond rupture process was repeated, and it was observed that the frequency remained almost unchanged before and after the cleavage. This indicates that a bond rupture experiment was sufficient to remove the aptamer–magnetic beads binding LF, verifying the feasibility of the sensor.

(1) $$\Delta f=\frac{-2f_{ο}^{2}\Delta m}{A\sqrt{\rho_{q}\mu_{q}}}$$

(2) $$F=\frac{2}{7}mA{\left(2\pi f\right)}^{2}$$

(3) $$A=1.4QV_{D}$$

According to the Sauerbrey equation (Equation (1)), the sensitivity of the 60 MHz base frequency crystal is 0.00177 ng/(mm^2^·Hz). Utilizing the sandwich method, a 100 ng/mL LF solution was measured with a molecular bond crack sensing instrument. Following an 8 V voltage excitation, the frequency shift was 1180 Hz, corresponding to a loss of approximately 1.28 ng of magnetic bead–aptamer from the active surface. The shear force at the surface of the shear-vibrating quartz crystal is detailed in Equation (2). Let A represent the maximum displacement of the piezoelectric quartz crystal’s vibration (Equation (3), where Q is the quality factor of the quartz crystal vibration, Q = 1500; and V_D_ is the driving excitation voltage). By substituting into the formula, we calculate F = 0.0606 nN. Molecular-specific binding forces range from 10 to 200 pN [24], which indicates that the bond cleavage force is adequate to disrupt the bond between the surface aptamer II–magnetic bead complex and LF. The shear force correlates with the vibration frequency and excitation voltage of the piezoelectric quartz. By fine-tuning the excitation voltage at the resonant frequency of piezoelectric quartz crystals, various shear forces can be generated. It can facilitate the molecular bonding process on the crystal surface [17]. The experimental findings demonstrate that for a 60 MHz piezoelectric quartz crystal, an 8 V excitation voltage can induce the molecular bond rupture of the outer magnetic bead–aptamer II within the Au–aptamer I–LF–aptamer II (magnetic beads) affinity layer within 20 s. Post-detachment, the resonant frequency of the quartz crystal increases, which was corroborated by the surface electrochemical signals pre- and post-bond cleavage.

### 2.4. Optimization of the Experimental Conditions

**Figure 4:**
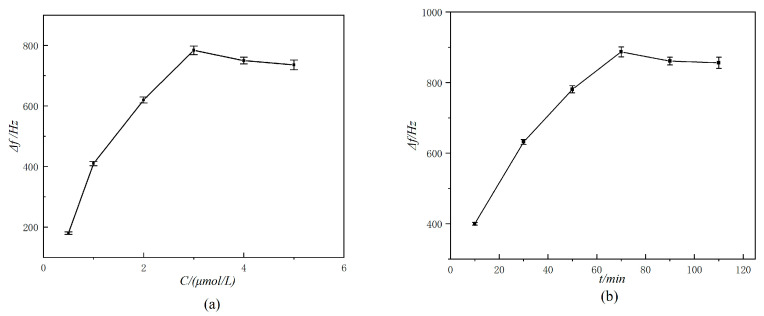

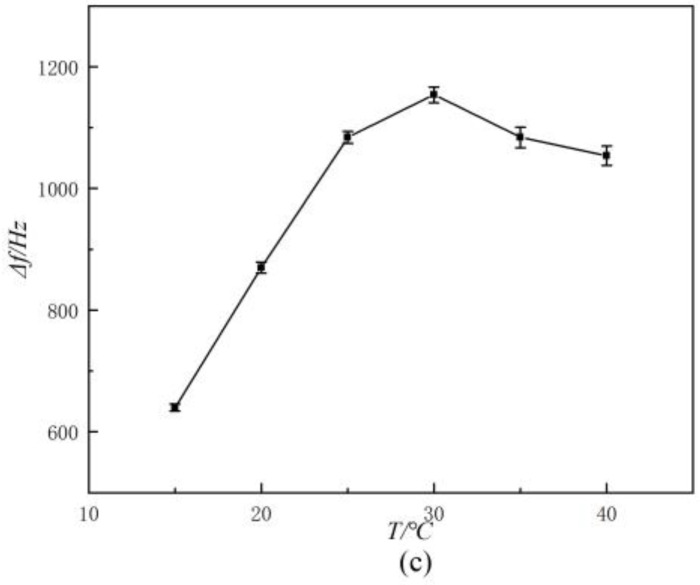
Effects of concentration of aptamer I (**a**), reaction time (**b**), and reaction temperature (**c**) on the frequency of bond rupture.

The detection performance of the sensor is significantly influenced by aptamer concentration, reaction time, and the reaction temperature. As shown in Figure 4a, Δ*f* increases with the concentration of the LF aptamer, reaching a peak at 3.0 μmol/L. Therefore, the optimal aptamer concentration was determined to be 3.0 μmol/L. In Figure 4b, the Δ*f* of the sensor initially increases with the total reaction time and stabilizes after 70 min. Thus, the optimal reaction time was set at 70 min. Figure 4c illustrates that as the reaction temperature increases, the binding efficiency of the aptamer to LF also increases, which leads to a higher Δ*f* of the sensor. However, beyond 30 °C, Δf begins to decrease, making 30 °C the optimal reaction temperature.

### 2.5. Performance of the Aptamer QCM Sensor

**Figure 5:**
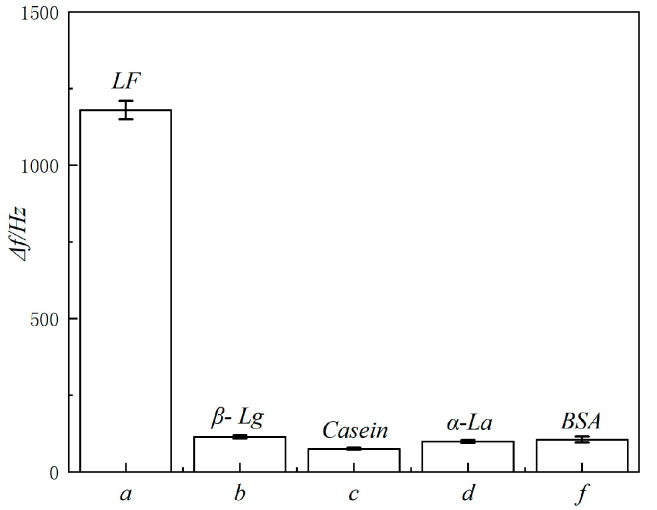
Specificity test of the QCM biosensor: (**a**) 100 ng/mL LF; (**b**) 100 ng/mL β-Lg; (**c**) 100 ng/mL Casein; (**d**) 100 ng/mL α-La; (**f**) 100 ng/mL BSA.

The sensor was used to detect the response signals of LF, β-lactoglobulin (β-Lg), Casein, α-lactalbumin (α-La), and bovine serum albumin (BSA) at 100 ng/mL, respectively. As shown in Figure 5, the QCM difference frequency signal values for detecting β-Lg, casein, α-La, and BSA are all small, which are 9.66%, 6.36%, 8.39%, and 8.90% of the difference frequency signal values for detecting LF, respectively, indicating that other protein components in the sample will not interfere with the sensor’s detection of LF. The results show that this method has good specificity.

**Figure 6:**
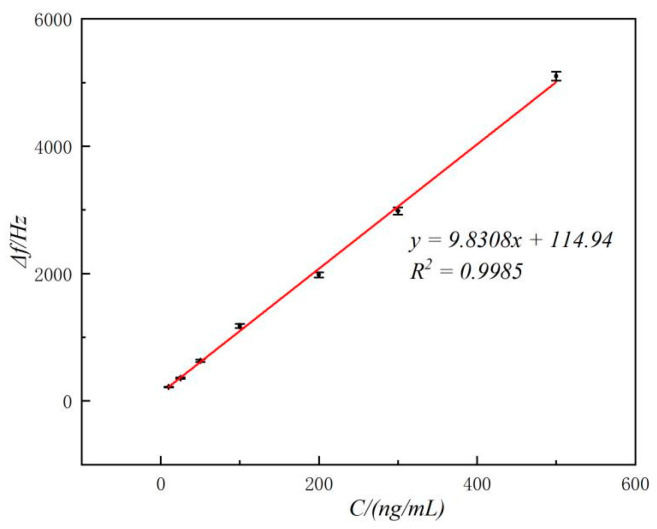
Calibration curve for the biosensor for detecting LF.

LF at different concentrations were examined under the best experimental conditions. The frequency change of the sensor increases with the concentration of LF, as depicted in Figure 6. This observation suggests that a higher amount of aptamer II–magnetic beads and LF are bound to the crystal surface, resulting in an increased release of aptamer II–magnetic beads during bond cracking. Δf increases linearly with increasing LF concentration, and the linear regression equation is Δ*f* (Hz) = 114.94 + 9.8308 *C_LF_* (ng/mL) (*R*^2^ = 0.9985), with the detection limit (3σ) of 8.2 ng/mL. Compared with other reported detection methods for LF (Table 1), this method has better sensitivity, a lower detection limit, and a shorter detection time, which is more suitable for the detection of bulk samples.

### 2.6. Analysis of the Actual Samples

Accurately measure 2 g (±0.01 g) of infant milk powder and dissolve it in approximately 25 mL of water at 45–50 °C. Transfer the mixture to a 150 mL conical flask and apply ultrasonic oscillation for at least 10 min to ensure complete dissolution. Let the solution stand for 5 to 10 min then cool to room temperature. Once at room temperature, adjust the pH to 5.3 ± 0.1 using acetic acid and transfer the solution to a 50 mL volumetric flask. Rinse the conical flask repeatedly with water, merge the washing solution into a 50 mL volumetric flask, make up to the mark with water, mix well, take a certain amount of sample solution, centrifuge for 10 min (5000 r/min), collect the supernatant, and pass it over 0.22 μm water system filter, which is the sample determination liquid. Dilute the sample measuring solution 100 times with 0.1 mol/L PBS (pH = 7.4) and then add different concentrations of LF to the diluted sample for detection [30]. As shown in Table 2, the recovery rate of this method ranges from 97.2 ± 4.4% to 106.0 ± 3.0%, and the RSD is less than 5%, indicating that the aptamer sensor has good practicality for analyzing LF in actual samples.

## 3. Materials and Methods

### 3.1. Instruments and Reagents

The electrochemical analyzer (CHI600D) was purchased from Shanghai Chenhua Instrument Co., Ltd., (Shanghai, China). The constant temperature mixer (HNMTH-100) was purchased from Shanghai Hannuo Instrument Co., Ltd., (Shanghai, China). The potential tester (MalvernNano-ZS) was purchased from Malvery Instruments Ltd., (Malvern, UK). The target analyte LF (CAS:146897-68-9, purity > 98%) was purchased from Sigma-Aldrich (St. Louis, MO, USA). The 1-ethy1-3-(3-(dimethylaminopropyl)carbodiimide (EDC) and N-hydroxy succinimide (NHS) were obtained from General Electric Company (Boston, MA, USA). The 6-Mercapto-1-hexanol (6-MCH) was purchased from Sigma-Aldrich (USA). The magnetic beads (-COOH, 200~300 nm) was purchased from Shanghai Maclean Reagent Company (Shanghai, China). Morolino ethanesulfonic acid (MES) and 3-(2-formyl) phosphonic hydrochloride (TCEP) were obtained from Shanghai Biotechnology Bioengineering Company (Shanghai, China). The aptamer I (5-SH(CH_2_)_6_-TGGTGCTGCCCCTAGTCTCCGGCTGCTTCTTGG-3) [8] and the aptamer (5-SH(CH_2_)_6_-AGGCAGGACACCGTAACCGGTGCA TCTATGGCTACTAGCTTTTCCTGCCT-3) [31] were synthetic and developed by Shanghai Biotechnology and Biological Engineering Company (Shanghai, China). Infant formula was purchased from a local supermarket (Shanghai, China).

### 3.2. QCM Molecular Bond Rupture Biosensing System

This study employed a custom-designed QCM biosensor for the detection of molecular bond fractures. The fundamental resonance frequency of a 60 MHz gold-plated quartz crystal was established using a Pierce oscillation circuit in conjunction with a subtraction frequency circuit system. The piezoelectric quartz crystal was interfaced with a high-frequency amplitude modulation module via a relay switch. This excitation module, controlled by a microcontroller, generated the 60 MHz quartz resonance frequency utilizing the DDS9854 digital signal generator, achieving an amplitude voltage peak of up to 8 V. Following 20 s of sinusoidal wave excitation at varying voltages, the differential frequency signal value from the piezoelectric quartz crystal was subsequently measured within the oscillation circuit.

### 3.3. Experimental Method

#### 3.3.1. Subsubsection

The magnetic bead–aptamers were synthesized using the method described in the referenced study [31]. A total of 50 μL of the magnetic bead solution was introduced into a 1.5 mL EP tube, and 50 mmol/L MES buffer (pH 6.0) was added to clean the beads; then, the EDC and NHS solutions was used to activate the carboxyl groups with 30 min. Magnetic separation and cleaning are performed; then, 10 μL of aptamer II (100 μmol/L) was added, and the mixture was incubated at room temperature with shaking for 6 h. After incubation, the magnetic separation and cleaning process was repeated to eliminate unbound aptamer. Finally, the aptamer-bound magnetic beads were resuspended in 1 mL of PBS (10 mmol/L) buffer and stored at 4 °C for future use.

#### 3.3.2. Modification of the QCM Crystal-Surface Aptamer

QCM crystals were treated with Piranha solution before use then washed with water and dried with nitrogen (N_2_). A 10 μL solution of aptamer I (3 μmol/L), reduced by TCEP, was added dropwise onto the surface of the crystal and incubated overnight. The crystals were then washed with water, followed by the addition of 10 μL MCH solution (1 mmol/L) and incubated for 60 min to seal the remaining sites. Finally, the crystals were rinsed with water and dried with nitrogen (N_2_).

#### 3.3.3. Detection of LF

The modified aptamer I crystal was placed in the detection cell, and 10 μL LF solution (100 ng/mL) was added. The reaction cell was shaken in a constant temperature mixer at 30 °C for 70 min and then carefully rinsed with water. A 10 μL aptamer II–magnetic ligand solution (100 μmol/L) was introduced and shaken in a constant-temperature mixer at 30 °C for 70 min. Finally, the obtained crystals were rinsed with water and dried with nitrogen (N_2_). After the dried crystals were placed in the QCM detection cell, the QCM sensor was activated and switched to amplitude modulation mode. The frequency change was observed and recorded.

#### 3.3.4. Electrochemical Measurement

Cyclic voltammetry was performed using a QCM crystal as the working electrode, an Ag/AgCl electrode as the reference electrode, and a platinum wire electrode as the counter electrode. The experiments were conducted in KCl solutions (100 mmol/L) containing K_3_Fe(CN)_6_–K^4^Fe(CN)_6_ (5 mmol/L). The scanning voltage range was set from −0.2 V to 0.6 V, with a scan rate of 0.1 V/s.

## 4. Conclusions

A piezoelectric quartz crystal-induced bond rupture aptamer biosensor was successfully developed for the detection of LF by utilizing adapter magnetic bead amplification signals. The mass response signal of the amplitude-modulated molecular bond rupture QCM biosensor was straightforward, using a high frequency of 60 MHz with high mass sensitivity. In the solution, the surface-modified molecular probe, specifically binding to the target substance, was introduced. By increasing the resonant excitation voltage of the piezoelectric quartz in the gas phase, the molecular bond cleavage in the surface binding layer was promoted. It can quickly obtain the quality response signal and significantly reduce the detection time. The amplitude-modulation bond crack QCM sensor is simple to operate, with high sensitivity and good specificity, which is suitable for the detection of large quantities of samples.

## Figures and Tables

**Table 1:** Comparison of the proposed method and other methods reported in the literature for the detection of LF.

Detection Method	Matrix	LOD	Req. Time	Ref.
HPLC	Papap milk, infant milk powder	0.25 μg/mL	Extraction of >2 h; Detection for 15 min	[25]
LC-MS/MS	Infant milk formula	0.16 mg/100 g	Enzyme hydrolysis > 8 h; detected for 5 min	[26]
AES	Pharmaceuticals and infant milk formula	0.027 μg/mL	>0.5 h	[27]
ELISA	Milk, curd, cream, and whey	0.062 μg/mL	2.5 h	[10]
FP assay	Milk powder	1.25 pM	0.5 h	[28]
CDFS	Not mentioned	0.776 μg/mL	Prepared for >24 h; Detection was performed for <10 min	[29]
QCM	Milk product	8.2 ng/mL	Batch reaction, and tested for <5 min	This work

**Table 2:** Detection results of LF in milk powder samples via the proposed molecular bond rupture QCM sensor testing.

NO.	Added	Blank	Found	Recovery	RSD
	(ng/mL)	(ng/mL)	(ng/mL)	(%)	(%, *n* = 3)
1	20	185.1 ± 3.7	206.3 ± 4.3	106.0 ± 3.0	4.4
2	50		237.5 ± 2.9	104.8 ± 2.6	4.9
3	100		287.5 ± 5.1	102.4 ± 3.8	3.5
4	300		384.5 ± 6.1	99.7 ± 3.3	4.2
5	500		671.2 ± 8.8	97.2 ± 4.4	2.9

## Data Availability

Data will be made available on request.
